# Influence of Effective Microorganisms on Some Biological and Biochemical Aspects of *Spodoptera littoralis* (Boisduval) (Lepidoptera: Noctuidae)

**DOI:** 10.3390/life12111726

**Published:** 2022-10-28

**Authors:** Mohamed S. Zayed, El-Kazafy A. Taha, Fatma H. Hegazy, Bander Albogami, Ahmed Noureldeen, El-Said M. Elnabawy

**Affiliations:** 1 Department of Pesticides, Faculty of Agriculture, Damietta University, Damietta 34517, Egypt; 2 Department of Economic Entomology, Faculty of Agriculture, Kafrelsheikh University, Kafrelsheikh 33516, Egypt; 3 Department of Plant Protection, Faculty of Agriculture, Tanta University, Tanta 31527, Egypt; 4 Department of Biology, College of Science, Taif University, Taif 21944, Saudi Arabia

**Keywords:** *Spodoptera littoralis*, immature stages, effective microorganisms, antifeedant effect

## Abstract

The cotton leafworm, *Spodoptera littoralis* (Bosid.), is a major pest in African and Asian nations that attacks a wide variety of host plants. This study was conducted to assess the effectiveness of effective microorganisms (EMs) on the biological and physiological features of *S. littoralis* larvae. Five concentrations (100, 200, 300, 400, and 500 ppm) of EMs were tested. Antifeedant activity, food consumption index, the efficiency of converting digested food, the efficiency of converting ingested food, relative growth rate, and approximate body tissue of the fourth larval instar of *S. littoralis* were determined. Moreover, carbohydrate enzyme activities (amylase, trehalose, and invertase), total protein, and total lipids of *S. littoralis* larvae were measured to elucidate the mode of action of the tested agent in the *S. littoralis’s* larval stage. The EMs at 500 ppm had a substantial impact on antifeedant activity, nutritional indices, egg deposit reduction, and hatchability in *S. littoralis* during the five days. All concentrations interrupted *S. littoralis*’s life cycle and developmental phases. Furthermore, all concentrations were quite useful in lengthening the developmental stages of *S. littoralis*. In addition, Ems affected the biochemical activities of larvae, leading to disturbances in carbohydrate, lipid, and protein levels. From this study, EMs can be used as a bioinsecticide alternative to traditional insecticides against *S. littoralis* and may be compatible with integrated pest management approaches.

## 1. Introduction

*Spodoptera littoralis* is one of the most dangerous insect crop pests. It can feed and attack several economic crops such as cotton, rice, maize, alfalfa, tomato, potato, ornamental plants, and orchard trees [1] and causes significant losses with respect to food and non-food crops worldwide. Moreover, it developed resistance to a variety of pesticides that are routinely employed in Egypt in chemical pest management operations on a variety of crops [2].

The increasing use of conventional pesticides led to numerous serious problems, such as environmental contamination, the annihilation of natural enemies, and insect resistance to different insecticides. Globally, 954 pest species were found to have developed pest resistance over time [3]. Thus, it is essential to create alternative or extra techniques [4]. Recently, many researchers focused on diverse strategies for insect pest control, including employing plant inducers to improve plants’ systemic resistance to some insect pests [5] and utilizing plant essential oils to control some insect pests [6]. Furthermore, Nasr et al. [7] reported that gamma irradiation can effectively control stored grain pests. As a result, more compounds are created by using recreations of naturally occurring biological poisons that have a range of biological impacts. On the other hand, previous studies showed how flowering plants and organic fertilizers improved the number of natural enemies, which in turn inhibited several related insect pests [8,9,10]. In addition, Shawer et al. [11] and Taha et al. [12] proved that *Trichogramma Evanecsens*’s fitness has been affected by emergence times and cold storage durations.

Biopesticides are a wide class of biochemicals and microbial insecticides produced from microorganisms and other natural origins [13,14]. These include naturally occurring pest control agents (biochemical pesticides), microorganisms that control pests (microbial pesticides), and pesticides produced by plants with genetically modified organisms (plant-incorporated protectants) [15,16]. The use of bioinsecticides to slow or stop the natural transition from juvenile to adult stages is one of the most promising microbial pesticide alternatives [17,18,19]. The use of *Bacillus thuringiensis* in integrated pest management (IPM) has been reported to be effective against larvae of many lepidopteran insects [20]. Additionally, EMs could be a beneficial component of an integrated pest management strategy for managing *S. littorals* [21]. The employment of microorganisms in synthesis is ecologically acceptable and compatible with green chemistry concepts [22].

Effective microorganism technology was founded in the 1970s at the University of Ryukyus, Okinawa, Japan. Studies indicated that the use of effective microorganisms (EM) is useful in several industries, such as livestock, agriculture, landscaping, gardening, bioremediation, composting, septic tank cleaning, algal management, and home applications [23]. The practical application of EMs was created by Prof Teuro Higa, who spent a long period during his scholarly career on isolating and choosing various microorganisms for their advantageous effects on soils and plants. Additionally, Prof. Teuro Higa identified microorganisms that can coexist in mixed cultures and are compatible physiologically. The positive effects of each of these cultures are substantially amplified when they are combined with the environment in a synergistic way [24]. The term “effective microorganisms” means a microbial inoculant that contains numerous varieties of beneficial microbes that are found in nature [25]. According to research conducted in the 1970s by Teuro Higa, a mixture of about 80 distinct microorganisms has the ability to convert the decomposition of organic matter back into a process that promotes life. Moreover, the use of EM will improve soil and irrigation water. It can be used to produce organic sprays for enhancing photosynthesis and reducing pests, diseases, and insects [26]. EMs are a combination of various helpful microorganisms that have shown effectiveness in insect management [5]. This might be due to their capacity to produce insect-repelling esterases, defensive enzymes, and release hydrolysis acids [27,28]. Arbuscular mycorrhizal fungi, plant-growth-promoting rhizobacteria, and plant-growth-promoting fungi are three different types of helpful microbes that can improve plant development and resistance to biotic and abiotic stresses [29]. Some previous studies evaluated the insecticidal activity of biocontrol agent EMs against *Pectinophora gossypiella* [30], *S. littorals* [21], and *Earias insulana* [31] and as an acaricide against *Tetranychus urticae* [32], and they found that EMs had significant effects on the tested pests. The usage of EM in fermented plant extracts on cucumbers cultivated organically decreased *Diaphania nitidalis* infestation [33]. Due to competitive and/or antagonistic behaviours of the microbes contained in the EM inoculant, diseases and pests are reduced or controlled [34]. Additionally, Zayed et al. [5] indicated that some bacteria have the ability to emit a range of volatile and nonvolatile compounds that stimulate plant development via various pathways, or they may secrete some poisons that reduce *Bemisia tabaci* populations.

Based on Siegel et al. [35] and Abdullah et al. [36], EMs include lactic acid bacteria, photosynthetic bacteria, yeasts, actinomycetes, and fermenting fungi, which have been reported as plant-growth inducers [5,37]. Additionally, Moon et al. [38] described the functions of different microbes of EMs. Lactic acid bacteria (*Lactobacillus plantarum*, *Lactobacillus casei*, and *Streptococcus lactis*) produce lactic acid and the breakdown of lignin and cellulose [38]. Photosynthetic bacteria (*Rhodopseudomonas palustris* and *Rhodobacter sphaeroides*) play a potent role in antioxidant synthesis; N2 and CO2 fixation; and amino acid nucleic acids, bioactive substances, and sugars syntheses [38]. Yeasts (*Saccharomyces cerevisiae* and *Candida utilis*) are responsible for bioactive substance synthesis (hormones and enzymes) [38]. Actinomycetes (*Streptomyces albus* and *S. griseus*) play an effective role in antimicrobial activities. Fungi (*Aspergillus oryzae* and *Mucor hiemalis*) support the production of alcohol, esters, and antimicrobial substances [38]. Thus, we may hypothesize that EMs have growth regulatory impacts against the *S. littoralis* larvae. The main purpose of this study was to assess the antifeedant impact of EMs on the fourth larval instar of *S. littoralis*. Moreover, this study estimated the impact of EMs on several biological and physiological features of *S. littoralis* larvae.

## 2. Materials and Methods

### 2.1. Rearing Technique of S. littoralis

The 1st instar larvae of the cotton leafworm *S. littoralis* (Bosid.) were received from the Cotton Leaf Worm Division, Plant Protection Research Institute, Agricultural Research Center. Before any treatment, larvae were grown on castor bean leaves (*Ricinus communis* L.) using the method reported by El-Defrawi et al. [39]. The pupae were collected and placed in clean jars with damp sawdust at the bottom to serve as a pupation site. A 10% sugar solution was given to the adults. All stages of *S. littoralis* were reared and tested at 27 °C and 75% R.H.

### 2.2. The Tested Agent

EMs were delivered by the Ministry of Agriculture, Cairo, Egypt. This EM formulation comprised approximately 80 different species of beneficial microorganisms that were cultured in a specific medium and produced locally in Egypt under the supervision of the Japanese EMRO (EMs Research Organization). This technology was established and developed by Prof Teuro Higa at the University of Ryukyus, Okinawa, Japan, in the 1970s. The microorganisms included lactic acid bacteria (*Lactobacillus casei*, *Streptoccus lactis* and *L. plantarum*), photosynthetic bacteria (*Rhodopseudomonas palustris* and *Rhodobacter sphaeroides*), Actinomycetes (*Streptomyces albus* and *S. griseus*), fermenting fungi (*Mucor hiemalis* and *Aspergillus oryzae*), and yeasts (*Candida utilis* and *Saccharomyces cerevisiae*). The formulation’s pH was 3 and it was stored in a refrigerator at zero ºC. It was diluted in water at 100, 200, 300, 400, and 500 ppm.

### 2.3. The EMs Preparation

When first accessed, EMs are in a dormant state and needs to be activated by adding 100 g of pure cane sugar to 1 L of dormant EMs. The formulation of EMs was kept in a clean and airtight plastic container that has no air remaining in it. The pH of the EMs is also important and it should be less than 4 [40].

### 2.4. Antifeedant Technique

The pre-starved (24 h) larvae were allowed to feed on the treated leaf discs for 5 days [41]. One hundred 4th instar larvae were divided into 4 replicates (25 larvae for each). Castor bean leaves were dipped for 5 min at each specified concentration. Additionally, as a control, the leaves were only dipped in water. Then, the leaves were allowed to dry under lab conditions. Then, the newly moulted 4th instar larvae of *S. littoralis* were placed in a glass container (18 cm in depth and 15 cm in diameter) and covered with muslin and fed on the treated leaves for 5 days. The treated leaves were replaced daily by new treated ones for five days. After that, the larvae are allowed to feed on untreated ones until pupation or death. Moreover, to assess larval mortality, the jars were checked each day and the corrected larval mortality was calculated by using Abbott’s formula [42]. Every day, the new and old (the uneaten area of the leaf discs) castor bean leaves, feces, and larvae in each rearing jar were weighed and recorded daily. The amount of consumed food was calculated by subtracting the dry weight of the food remaining at the end of the trial from the total weight of the food supplied. The percentage of feeding inhibition was calculated after 5 days using the Abivardi and Benz [43] equation (Equation (1)):

(1) $$\%\;\mathrm{Antifeedant}\;\mathrm{activity}\;=\frac{C-T}{C\;}\times 100$$

where 

C = weight of diet consumed in the untreated meal (control);

T = weight of diet consumed in the treated meal.

Food consumption, the efficiency of conversion of digested food, the efficiency of conversion of ingested food, relative growth rate, and approximate digestibility were determined using the equations developed by Waldbauer [44] and Senthil-Nathan and Kalaivani [45] as follows:

Consumption index (CI) = E/TA

The efficiency of conversion of digested food (ECD) = 100 × P/(E – F)

The efficiency of conversion of ingested food (ECI) = (P/E) × 100

Relative growth rate (RGR) = P/T

Approximate digestibility (AD) = [(E – F)/E] × 100

A = Mean dry weight of larvae during the experimental period.

P = Dry weight gain of larvae.

E = Dry weight of food eaten.

F = Dry weight of feces produced.

T = Duration of the experimental period.

### 2.5. Biological Aspects

Survived larvae were transferred to jars containing fresh untreated leaves and were observed daily to determine the weight, normal, malformation, and duration of larvae and pupae and malformed and newly emerged adults. Two females and one male of the resulting adults were placed together in a wooden box to maximize successful mating; they were provided with a piece of cotton soaked in 10% sugar solution as a source of food for each of the 5 concentrations and the control. The period from adult emergence until adult death for males and females was calculated to determine adult longevity. The number of eggs per female was counted to determine fecundity. Three patches having not less than 100 eggs were collected during 5 days of oviposition and incubated under laboratory conditions until hatching, and then the percentage of eggs’ hatchability was calculated. The reduction percentage of deposited eggs was calculated for each treatment according to Mohamed [46] as follows (Equation (2)).

(2) $$\%\;\mathrm{Reduction}=\frac{\mathrm{Number}\;\mathrm{in}\;\mathrm{treatment}-\mathrm{Number}\;\mathrm{in}\;\mathrm{control}}{\mathrm{Number}\;\mathrm{in}\;\mathrm{control}}\;\;\times \;100$$

### 2.6. Enzymes Assay

After 6 h of starvation, a homogenate of the 4th instar larvae treated with the tested EMs concentrations was prepared. According to El-Doksh [47], each sample was ground in a mortar to obtain the fine powder, suspended in an ice solution of 0.25 M sucrose, and centrifuged at 3000 rpm. The supernatants were used to assess the activity of carbohydrates enzymes (amylase, trehalose, and invertase), total protein, and total lipids using methods described by Ishaaya and Swirski [48], Henry [49], and Schmit [50], respectively.

### 2.7. Statistical Analysis

All data were tested for normal distribution using the Shapiro–Wilk normality test, which revealed that the data had a normal distribution. As a result, the analysis was performed using the original data. One-way ANOVA was used to assess all aspects. Tukey’s HSD post hoc test was used to assess differences among various parameter means by using the Costat system for Windows, Version 6.311 [51].

## 3. Results

The antifeedant activity of certain EMs at different concentrations on *S. littoralis* fourth larval instars is shown in Table 1. The 500 ppm of EMs exhibited a significantly higher antifeedant effect compared with the other concentrations, while 100 ppm was the least effective concentration. In general, the antifeedant effect was gradually enhanced by increasing the EM’s concentration during the feeding of larvae on treated leaves. Furthermore, antifeedant activities differed among different concentrations at the first day (F4,10 = 3047.79, *p* < 0.01), the 2nd day (F4,10 = 2186.82, *p* < 0.01), the 3rd day (F4,10 = 1452.40, *p* < 0.01), 4th day (F4,10 = 3339.25, *p* < 0.01) and 5th day (F4,10 = 1075.81, *p* < 0.01).

Data presented in Table 2 show that all membered concentrations showed a significant decrease in the food consumption index (CI) (F5,12 = 110.32, *p* < 0.01), the relative growth rate (RGR) (F5,12 = 300.62, *p* < 0.01), approximate digestibility (AD) (F5,12 = 359.11, *p* < 0.01), the efficiency of converting ingested food (ECI) (F5,12 = 138.49, *p* < 0.01), and converting digested food (CD) (F5,12 = 333.81, *p* < 0.01) into body tissues compared with the control. On the other hand, the ability of larvae to utilize food for growth was measured by the approximate digestibility (AD), which measures the digestion of food ingested by larvae. In general, it was observed that 500 ppm of EMs was more effective in all tested parameters compared with the control, while 100 ppm was the least effective treatment in all tested parameters.

Data in Table 3 indicate the effect of EMs at different concentrations on the reduction percentage of the number of deposited eggs/female of *S. littoralis.* The parts-per notation at 500 ppm caused the highest reduction (72.78%) in the number of deposited eggs/female, while 100 ppm caused the least amount of reduction (33.06%) in the number of deposited eggs/female. The tested agent had a modest effect on rapid egg deposition (after 1–2 days). As a consequence, the tested agent had the greatest late egg deposition impact (after 4–5 days). Moreover, the reduction percentages of the number of deposited eggs/female significantly differed based on different concentrations of EMs on the 1st day (F4,10 = 20.75, *p* < 0.01), the 2nd day (F4,10 = 91.09, *p* < 0.01), the 3rd day (F4,10 = 381.61, *p* < 0.01), the 4th day (F4,10 = 168.04, *p* < 0.01), and the 5th day (F4,10 = 298.73, *p* < 0.01).

**Figure 1:**
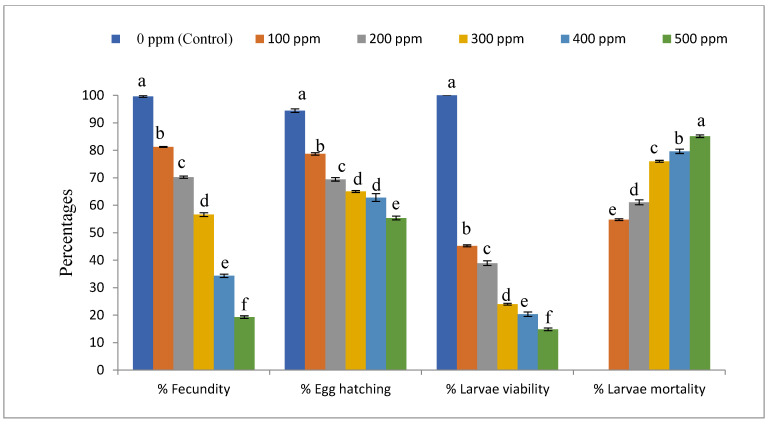
Effect of EMs on some biological aspects of *Spodoptera littoralis*. Different letters above the bars on the same parameter indicate a significant difference (*p* < 0.05) according to Tukey’s test.

**Figure 2:**
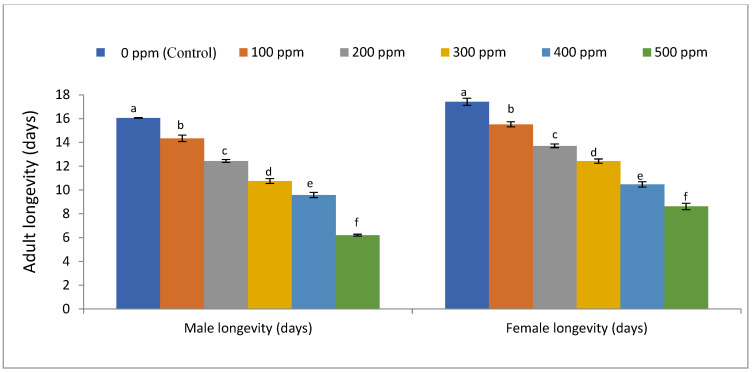
Effect of EMs on the longevity of *S. littoralis*. Different letters above the bars on the same sex indicate a significant difference (*p* < 0.05) according to Tukey’s test.

Data in Figure 1 and Figure 2 show that all concentrations of EMs had significant effects on the tested biological aspects of *S. littoralis*, fecundity (F5,12 = 3243.87, *p* < 0.01), egg hatchability (F5,12 = 198.22, *p* < 0.01), larvae viability (F5,12 = 1966.51, *p* < 0.01), larvae mortality (F5,12 = 251.89, *p* < 0.01), and male (F5,12 = 271.51, *p* < 0.01) and female (F5,12 = 120.94, *p* < 0.01) longevity of *S. littoralis*. Moreover, the 500 ppm level was the most effective treatment for reducing fecundity, egg hatchability, larvae viability, larvae mortality (Figure 1), and male and female longevity (Figure 2), while the 100 ppm level was the least effective treatment on fecundity, egg hatchability, larvae viability, larvae mortality, and male and female longevity of *S. littoralis*.

Data in Table 4 refer to the effect of different concentrations of the tested agent against the developmental stages (larva, pupa, and adult) of *S. littoralis.* For the larval and pupal stages, all concentrations affected the weight, normal, malformed, and duration of *S. littoralis.* The 500 ppm level was the most effective treatment, while 100 ppm was the least effective treatment. Additionally, there were large differences in the weight (F5,12 = 36.37, *p* < 0.01), normal percentages (F5,12 = 3517.96, *p* < 0.01), malformed percentages (F5,12 = 677.88, *p* < 0.01), and duration (F5,12 = 389.55, *p* < 0.01) of the *S. littoralis* larvae treated with different concentrations of EMs. Moreover, for the pupal stage, the weight (F5,12 = 2407.92, *p* < 0.01), normal percentage (F5,12 = 235.71, *p* < 0.01), malformed percentage (F5,12 = 586.19, *p* < 0.01), and duration (F5,12 = 403.37, *p* < 0.01) were statistically different based on different concentrations of EMs. For the adult stage, the highest value (80.89 adults) of a malformed adult was recorded at 500 ppm, while the highest value (48.68 adults) of an emerged adult was recorded at 100 ppm. The different concentrations of EMs had significant differences in malformed percentages (F5,12 = 598.82, *p* < 0.01) and emergence percentages of adults (F5,12 = 598.82, *p* < 0.01).

The data listed in Table 5 show that treatments with 500 ppm EMs resulted in the greatest reduction in trehalose and amylase followed by 400 and 300 ppm, whereas other concentrations increased trehalose activity by 100 and 200. Treatment with EMs resulted in the greatest reduction in amylase at 500, 400, 300, and 200 ppm. In contrast, treatment with 100 ppm boosted the activity of the amylase enzyme. Except for 100 ppm, all concentrations of invertase rose dramatically in invertase activity. In terms of total protein and fat content, according to the toxicity data, the results indicated that all concentrations of the tested chemical reduced the total protein and lipid levels. The different concentrations of EMs had significant impacts on the following enzyme activities, trehalose (F5,12 = 690.29, *p* < 0.01), amylase (F5,12 = 76.95, *p* < 0.01), invertase (F5,12 = 255.22, *p* < 0.01), total protein (F5,12 = 48.25, *p* < 0.01), and total lipids (F5,12 = 36.01, *p* < 0.01).

## 4. Discussion

The assessment of the mode of action of EMS against *S. littoralis* larvae may be a complex process that deserves further investigation. For EMs, we hypothesize that some microbes can emit a range of volatile and non-volatile compounds that affect *S. littoralis*. Our results indicated that the EM’s concentrations had antifeeding activities against the fourth instar larvae of *S. littoralis*. The combined influence of repellents, bad taste, enzyme inhibition, and non-enzymatic characteristics such as total protein and total lipids may account for the enhanced antifeedant activity of all concentrations. Moreover, the studied agent’s fast antifeedant activity (after 1–2 days) may be ascribed to its repelling impact or its unpleasant taste for the larvae. As a result, the larvae did not eat at first, but after starving, the larvae began consumption. The tested compound’s late antifeedant activity (after 4–5 days) may be related to the inhibitory impact of the tested agent on digestive enzymes, which allowed larvae to consume at first and thereafter cease feeding. EMs concentrations had a significant impact on the food consumption index, the relative growth rate, approximate digestibility, efficiency of converting ingested food, and converting digested food into body tissue. Our hypothesis agreed with those of Moon et al. [38], who reported that fungi (*Aspergillus oryzae* and *Mucor hiemalis*) support the production of alcohol, esters, and antimicrobial substances, which maybe inhibit the insect’s feed. Moreover, Senthil-Nathan et al. [45] found that avermectin (*Streptomycetes avermectins*) had remarkable antifeeding activity on *S. littoralis* larvae, accompanied by a reduced relative consumption index (CI) and relative growth rate (RGR). El-Malla and Radwan [41] found that growth rate (GR), CI, approximate digestibility (AD), the efficiency of conversions of either ingested (ECI) or digested (ECD) food to the body tissue of *S. littoralis* larvae fed on biopesticides decreased compared with the control except for another biopesticide spinosad treatment, which produced a slight increase in the same parameters. Ebeid and Gesraha [52] indicated that spinosad reduced food consumption, GR, ECI, and ECD in body tissues. The AD considerably increased by increasing the concentration of EMs; this may be due to some of these microbial mixtures secreting some digestive and hydrolytic enzymes, and this is agreed with by the authors of [27,28]. The observed variations in antifeedant activity levels of *S. littoralis* after EMs treatments might be caused by physiological or pathological changes. Moreover, EMs had a potential efficacy against *S. littoralis*, and this may be due to the presence of several microbial isolates in the formulation that have entomopathogenic activities via the production of some toxic secondary metabolites [35,36]. According to Isman [53], antifeedants have some physiological or toxic actions on insects depending on treatment concentrations. We suppose that some microbes can secrete a variety of volatile and non-volatile metabolites that promote plant growth via different pathways or may secrete some toxins that increase the antifeedant’s activity. Our hypothesis agreed with Fincheira and Andrés [37]; they proposed that the use of volatile organic compounds emitted by microorganisms will be useful as a technological innovation in agriculture. Some of these compounds are 2,3 butanediol, 3-hydroxy-2-butanone (acetoin), 2-pentylfuran, or dimethylhexadecylmine. Moreover, it has been demonstrated that several volatile organic compounds produced primarily by the fungus (2-octanone, 3-octanol, and 2–5-dimethylfuran) had insecticidal activity [54,55]. A key advantage of biological agents relative to chemical pesticides is their capacity to kill pests (functional response) and to reproduce at the expense of pests (numerical response), thereby giving some control over future pest generations. Moreover, Yasui et al. [56] revealed that an antifeedant is a pesticide that inhibits insects from eating while lingering near treated crops and starving to death.

Our findings revealed that the use of EMs concentrations against the fourth larval instar of *S. littoralis* results in a decrease in egg production, hatchability, and other biological aspects of reproductive capacities. Additionally, it negatively affects carbohydrate enzymes activities, total protein, and total lipid contents. This decrease in egg production, hatchability, and other biological aspects of reproductive capacity may be due to the breakdown of protein into amino acids, carbohydrates, and lipids, which helps supply energy for the insect in terms of egg deposition operations. The carbohydrate, protein, and lipid components supply insects with glucose and amino acids during the development stage, acting as an energy source for the formation of larval and adult tissues, particularly the cuticle. Moreover, changes in the protein content probably reflect the balance between synthesis, storage, transport, and degradation of structural and functional nutrients during ontogeny as well as the response to particular physiological conditions. Carbohydrates are required for the correct operation of male and female reproductive systems. Sugars are a key component of the reproductive glands in males, and the testes contain the majority of the reproductive system’s carbs and are required in the female system for vitellogenesis and the synthesis of the glycosaminoglycan found in the vitalized membrane and chorion. Vitellogenesis is the buildup of carbohydrate, lipid, and protein yolk within the egg for satisfying the structural and metabolic demands of the developing embryo [57]. Rather than being a biomarker of larval defense or immunological competence, protein content is one of the indications of larval health [58]. Moreover, protein leakage during intoxication can be caused by reduced body weight, protein conversion to amino acids, protein breakdown to release energy, or the tested extract’s direct effects on the cell’s amino acid transport system [59]. Our results indicated that the disturbance of carbohydrates and chitinase enzymes may be the cause of the high malformation rate of developmental stages (larvae, pupa, and adult). These results agree with those of Derbalah et al. [30], who revealed that the reduction in proteins, lipids, carbohydrate hydrolyses, and liver function enzymes by EMs caused inhibition and/or reduced chitin contents in larvae of *Pectinophora gossypiella* Saunders, as well as a reduction in reproductive potentiality. Moreover, Rawi et al. [59] found that the treatment of EMs mildly decreased the total protein. Moreover, many researchers reported the effect of bioinsecticides and insect growth regulators on the developmental stages of *S. littoralis* [60].

Our results indicated that the use of EMs had a significant effect on malformation. The high malformation rate may be due to the decrease in total protein, carbohydrates, and total lipids. These results agree with those of Osman et al. [21]; they observed a relationship between the high malformation rate in the developmental stages and a more significant decrease in total carbohydrates, total protein, and total lipid contents in the sixth instars of *S.* *Littoralis* treated with EMs. Keratum et al. [31] found that carbohydrates activities and total protein and total lipid contents decreased in the fourth instar larvae of spiny bollworm *Earias insulana* treated with EMs. Moreover, Zayed [32] found that EMs disrupted carbohydrates, total protein, and total lipids in *T. urticae*. Our findings, in parallel with similar results, were obtained by Abdel-Aal and Abdel-khalek [61]; EL-Shershaby et al. [62] and Kamel et al. [63] reported that *B. thuringiensis* caused a significant reduction in the protein content of *S. littorals* larvae. Furthermore, Assar et al. [64] found that the total protein content decreased in the house fly treated with insect growth regulators. Obtained results revealed that the reduction in protein-, lipid-, and carbohydrate-hydrolyzing enzymes caused reduced and inhibited chitin contents in the larvae of *S. littoralis* and caused inhibition in developmental stages and reproductive potentiality as egg deposition. All of these changes finally may be led to disrupted weight, malformation, duration, and finally the mortality of treated larvae, pupa, and adults. Relationships between the growth rate and protein content as well as enzyme activity provided strong evidence for the role of biochemical changes in insect mortality [65,66].

## 5. Conclusions

In summary, these findings showed that the use of EMs has a significant effect on *S. littoralis*. It had significant impacts on antifeedant activity, food consumption index, the efficiency of converting digested food, the efficiency of converting ingested food, and the relative growth rate. Moreover, it enhanced the reduction percentage of the number of deposited eggs/female of *S. littoralis*. Moreover, it affected the hatchability of *S. littoralis*. In addition, all concentrations interrupted *S. littoralis* ‘s life cycle and developmental phases. Thus, the EM has shown its potential application as an alternative control method to conventional insecticides for *S. littoralis* management and is compatible with IPM techniques.

## Figures and Tables

**Table 1:** The antifeedant activity of EMs against the 4th larval instar of *S. littoralis*.

EMs (ppm)	% Antifeedant Activity					Mean
	1st Day	2nd Day	3rd Day	4th Day	5th Day	
100.00	9.85 ± 0.30 ^e^	15.79 ± 0.04 ^e^	22.03 ± 0.21 ^e^	33.01 ± 0.37 ^e^	41.71 ± 0.86 ^e^	22.03 ± 0.38 ^e^
200.00	13.80 ± 0.33 ^d^	19.62 ± 0.30 ^d^	28.18 ± 0.36 ^d^	38.29± 0.86 ^d^	49.87 ± 0.43 ^d^	28.18 ±0.21 ^d^
300.00	19.46 ± 0.12 ^c^	23.16 ±0.16 ^c^	34.01 ± 0.40 ^c^	45.26 ± 0.32 ^c^	55.09 ±0.36 ^c^	34.01± 0.22 ^c^
400.00	21.41 ± 0.20 ^b^	37.56 ± 0.26 ^b^	48.30 ± 0.60 ^b^	60.51 ± 0.18 ^b^	68.22 ± 0.36 ^b^	48.29 ± 0.40 ^b^
500.00	34.29 ± 0.02 ^a^	45.01 ± 0.46 ^a^	56.52 ± 0.74 ^a^	66.39 ± 0.05 ^a^	78.52 ± 0.23 ^a^	56.52 ±0.16 ^a^

Values are the mean ± standard error. The means in each column followed by a different letter are statistically different at the 0.05 level.

**Table 2:** Effect of EMs on consumption and utilization of food of the fourth larval instar of *S. littoralis*.

EMs (ppm)	CI	RGR%	AD%	ECI%	CD%
0.00 (Control)	6.37 ± 0.15 ^a^	24.19 ± 0.11 ^a^	84.70 ± 0.09 ^f^	8.53 ± 0.06 ^a^	10.37 ± 0.04 ^a^
100.00	5.61 ± 0.16 ^b^	19.63 ± 0.57 ^b^	86.33 ± 0.40 ^e^	7.24 ± 0.11 ^b^	8.09 ± 0.15 ^b^
200.00	4.52 ± 0.10 ^c^	18.44 ± 0.06 ^c^	89.02 ± 0.14 ^c^	6.51 ± 0.15 ^c^	7.15 ± 0.09 ^c^
300.00	3.76 ± 0.09 ^d^	16.51 ± 0.12 ^d^	87.68 ± 0.28 ^d^	5.62 ± 0.15 ^d^	6.27 ± 0.13 ^d^
400.00	3.09 ± 0.04 ^e^	12.59 ± 0.13 ^e^	94.81 ± 0.38 ^b^	4.11 ± 0.06 ^e^	4.77± 0.06 ^e^
500.00	2.87 ± 0.02 ^f^	9.60 ± 0.16 ^f^	97.38 ± 0.03 ^a^	3.44 ± 0.16 ^f^	3.90 ± 0.06 ^f^

Values are the mean ± standard error. The means in each column followed by a different letter are statistically different at the 0.05 level. Consumption index (CI); relative growth rate (RGR) %; approximate digestibility % (AD); efficiency of converting ingested food % (ECI); efficiency of converting digested food % (CD).

**Table 3:** Reduction percentage of number of deposited eggs/female of *S. littoralis*.

EMs (ppm)	Reduction%					
	1st Day	2nd Day	3rd Day	4th Day	5th Day	Mean
100.00	1.40 ±0.94 ^e^	18.77 ± 2.17 ^e^	34.44 ± 0.74 ^e^	50.63 ± 0.24 ^d^	62.91 ± 0.36 ^c^	33.06 ± 0.59 ^e^
200.00	19.68 ±1.26 ^d^	33.67 ± 0.83 ^d^	42.36 ± 0.95 ^d^	59.65 ± 0.51 ^c^	69.62 ±0.94 ^b^	44.07 ± 0.44 ^d^
300.00	33.29 ±1.35 ^c^	43.25± 0.63 ^c^	52.36 ± 0.26 ^c^	65.44 ±1.81 ^b^	71.94 ± 1.96 ^b^	52.49 ± 0.96 ^c^
400.00	46.42 ±1.07 ^b^	53.45 ± 1.09 ^b^	57.19 ± 1.19 ^b^	67.39 ±1.31 ^b^	76.64 ±1.40 ^a^	59.66 ±0.63 ^b^
500.00	67.40 ±0.97 ^a^	71.07 ± 0.28 ^a^	72.32 ± 0.79 ^a^	75.64± 0.56 ^a^	78.53 ±0.50 ^a^	72.78 ± 0.11 ^a^

Values are the mean ± standard error. The means in each column followed by a different letter are statistically different at the 0.05 level.

**Table 4:** Some biological aspects for survived 4th larval instar of *S. littoralis*, treated by EMs.

EMs (ppm)	Larvae			
	Weight (mg)	% Normal	% Malformed	Duration
0.00 (Control)	60.58 ± 0.77 ^a^	100.00 ± 0.00 ^a^	00.00 ± 0.00 ^f^	14.37± 0.10 ^f^
100.00	60.50 ± 3.68 ^a^	55.73± 0.45 ^b^	44.27 ± 0.45 ^e^	22.07 ± 0.08 ^a^
200.00	51.91 ± 1.10 ^b^	42.85± 0.37 ^c^	57.14 ± 0.37 ^d^	20.33 ± 0.07 ^b^
300.00	44.48± 0.72 ^c^	33.27 ± 0.63 ^d^	66.73 ±0.63 ^c^	18.16 ± 0.20 ^c^
400.00	37.65 ± 0.59 ^d^	23.45± 0.38 ^e^	76.55 ±0.38 ^b^	16.81 ± 0.04 ^d^
500.00	30.16 ± 0.39 ^e^	18.15± 0.22 ^f^	81.85 ± 0.22 ^a^	15.62 ± 0.15 ^e^
	**Pupae**			
**EMs (ppm)**	**Weight (mg)**	**Normal %**	**Malformed %**	**Duration**
0.00 (Control)	375.22 ±0.48 ^a^	93.33 ±1.66 ^a^	6.67 ±1.67 ^f^	13.7 ± 0.12 ^a^
100.00	354.99 ±0.30 ^b^	64.18 ±0.93 ^b^	35.81 ±0.93 ^e^	8.82 ± 0.06 ^f^
200.00	344.75 ± 0.88 ^c^	47.71 ± 1.40 ^c^	52.29 ±1.4 ^d^	9.19 ± 0.06 ^e^
300.00	335.70 ±1.48 ^d^	34.45 ± 0.77 ^d^	65.5 ±0.77 ^c^	10.42 ±0.06 ^d^
400.00	320.51± 0.35 ^e^	19.77 ± 2.84 ^e^	76.89 ±0.58 ^b^	11.13 ±0.04 ^c^
500.00	303.88 ± 0.53 ^f^	14.90 ± 0.41 ^e^	85.09 ±0.40 ^a^	12.56 ±0.05 ^b^
	**Adult**			
**EMs (ppm)**	** Emergence % **	** Malformed % **		
0.00 (Control)	90.67 ± 0.67 ^a^	9.33 ±0.66 ^f^		
100.00	51.32 ±0.44 ^f^	48.68 ±0.40 ^a^		
200.00	56.6 ± 0.40 ^e^	43.4 ± 0.42 ^b^		
300.00	61.72 ±0.42 ^d^	38.28 ±0.42 ^c^		
400.00	75.63 ± 0.59 ^c^	24.37 ± 0.59 ^d^		
500.00	80.89 ± 0.44 ^b^	19.11 ±0.44 ^e^		

Values are the mean ± standard error. The means in each column that are followed by a different letter are statistically different at the 0.05 level.

**Table 5:** Carbohydrates enzymes activities, total protein, and total lipid contents in 4th instar larval *S. littoralis* treated by the serial concentration of EMs.

EMs (ppm)	Carbohydrates Enzymes			Total Protein (g/L)	Total Lipids (g/L)
	Trehalose (µ/L)	Amylase (µ/L)	Invertase (µ/L)		
0.00 (Control)	180.01 ± 0.32 ^c^	18.86 ± 0.26 ^b^	112.09 ± 0.62 ^e^	5.01 ± 0.10 ^a^	7.04 ± 0.03 ^a^
100.00	189.35 ± 0.78 ^a^	19.51 ± 0.23 ^a^	111.49 ± 0.00 ^e^	4.77 ±0.02 ^b^	6.65 ± 0.02 ^a^
200.00	185.72 ± 0.33 ^b^	17.48 ± 0.26 ^c^	115.84 ± 0.33 ^d^	4.25 ± 0.03 ^c^	6.36 ± 0.29 ^b^
300.00	170.37 ± 0.27 ^d^	16.38 ± 0.04 ^d^	119.73 ± 0.49 ^c^	4.14 ±0.26 ^cd^	6.30 ± 0.22 ^b^
400.00	152.33 ± 0.47 ^e^	15.16 ± 0.16 ^e^	124.83 ± 0.22 ^b^	4.01 ± 0.26 ^de^	5.52 ± 0.00 ^c^
500.00	145.94 ± 0.01 ^f^	14.51 ± 0.00 ^f^	127.67 ± 0.32 ^a^	3.95 ± 0.00 ^e^	4.09 ± 0.00 ^d^

Values are the mean ± standard error. The means in each column that are followed by a different letter are statistically different at the 0.05 level.

## Data Availability

All data generated or analyzed during this study are included in this published article.
